# Statins, cholesterol and cognition at the time of Alzheimer's disease diagnosis: A cross-sectional study from the Swedish registry for cognitive/dementia disorders

**DOI:** 10.1177/25424823251385903

**Published:** 2025-10-03

**Authors:** Bojana Petek, Minjia Mo, Hong Xu, Jakob Norgren, Minh Tuan Hoang, Marta Villa-Lopez, Henrike Häbel, Julianna Kele, Luana Naia, Silvia Maioli, Joana B Pereira, Milica Gregorič Kramberger, Bengt Winblad, Maria Eriksdotter, Juan-Jesus Carrero, Sara Garcia-Ptacek

**Affiliations:** 1 Division of Neurogeriatrics, Department of Neurobiology, Care Sciences and Society, 27106 Karolinska Institutet, Stockholm, Sweden; 2 Clinical Institute of Genomic Medicine, University Medical Centre Ljubljana, Ljubljana, Slovenia; 3 Faculty of Medicine, University of Ljubljana, Ljubljana, Slovenia; 4 Division of Clinical Geriatrics, Department of Neurobiology, Care Sciences and Society, 27106 Karolinska Institutet, Stockholm, Sweden; 5 Department of Medical Epidemiology and Biostatistics, Karolinska Institutet, Stockholm, Sweden; 6 Department of Medicine, School of Medicine, Universidad Complutense, Madrid, Spain; 7 Division of Neurology, Department of Medicine, University of Alberta Hospital, Edmonton, Alberta, Canada; 8 Medical Statistics Unit, Department of Learning, Informatics, Management and Ethics, 27106 Karolinska Institutet, Stockholm, Sweden; 9 Team Neurovascular Biology and Health, Clinical Immunology, Department of Laboratory Medicine, 27106 Karolinska Institutet, Stockholm, Sweden; 10 Neuro Division, Department of Clinical Neurosciences, 27106 Karolinska Institutet, Stockholm, Sweden; 11 Department of Neurology, University Medical Centre Ljubljana, Ljubljana, Slovenia; 12 Theme Inflammation and Aging, Karolinska University Hospital, Huddinge, Sweden; 13 Division of Nephrology, Department of Clinical Sciences, Karolinska Institutet Danderyd Hospital, Stockholm, Sweden

**Keywords:** Alzheimer's disease, cholesterol, dementia, drug repurposing, Mini-Mental State Examination

## Abstract

**Background:**

Evidence suggests statins may influence cognition in Alzheimer's disease (AD), but specific use patterns in AD patients remain unclear.

**Objective:**

To identify factors influencing statin use in AD and explore associations between statins, cholesterol, and cognition, evaluated with Mini-Mental State Examination (MMSE) at dementia diagnosis.

**Methods:**

A cross-sectional study using data from the Swedish Registry for Dementia and Cognitive Disorders (SveDem) and Stockholm Creatinine Measurements (SCREAM) from 2007 to 2018. Multivariable logistic regression examined associations between baseline characteristics and statin use, while linear regression analyzed relationships between statins, cholesterol levels, and MMSE scores.

**Results:**

We included 3074 AD patients (mean age 78.1 years; 59.4% women), of whom 1028 used statins (79.6% simvastatin, 20.4% atorvastatin). Patients with diabetes mellitus, ischemic heart disease, or stroke had greater odds of receiving statins. Older patients had slightly lower odds of receiving any statin at baseline (simvastatin use OR 0.98, 95% CI 0.97–0.99). Simvastatin users had 0.53 points higher MMSE on average at baseline compared to non-users of statins (se 0.23, p = 0.021). Higher low-density lipoprotein cholesterol (LDL-C), total cholesterol (TC) and high-density lipoprotein cholesterol (HDL-C) levels were associated with higher MMSE in non-users of statins, but not in statin users.

**Conclusions:**

Younger AD patients and those with cardiovascular disease were more likely to use statins. Simvastatin use was linked to higher cognitive scores at diagnosis. In non-users, higher LDL-C, TC, and HDL-C levels correlated with better baseline cognitive scores. Longitudinal studies are needed to investigate the effects of statins on cognitive decline in AD.

## Introduction

The global prescription rates of statins, also known as 3-hydroxy-3-methylglutaryl coenzyme A (HMG-CoA) reductase inhibitors, have been increasing over the past few decades. This trend reflects the widespread use of these medications for the prevention of cardiovascular and cerebrovascular diseases driven by expanding clinical guidelines, aging populations, evidence of efficacy and generic availability.^1–3^ Statins primarily work by reducing peripheral low-density lipoprotein cholesterol (LDL-C) levels.^4^ Additionally, statins have been shown to increase high-density lipoprotein cholesterol (HDL-C) levels by 5–15% through various mechanisms, though the clinical benefit of this effect remains unclear.^5,6^ Finally, statins have been associated with a modest decrease in triglycerides levels.^7^

Statins are commonly classified as lipophilic (e.g., simvastatin, atorvastatin) or hydrophilic (e.g., rosuvastatin, pravastatin),^8^ a distinction relevant to their potential central nervous system effects. Lipophilic statins are generally more likely to cross the blood-brain barrier (BBB), though other pharmacokinetic factors—such as molecular weight and transporter interactions—also play a role.^9^ Notably, simvastatin showed a higher BBB penetration (>25%), while atorvastatin, despite being lipophilic, had limited penetration (<5%), likely due to its size and structure.^9^

Besides cardiovascular effects, statins have attracted significant attention for their potential cognitive effects, both through lipid-lowering and their pleiotropic actions.^8,10,11^ Cholesterol plays a key role in brain function, influencing synaptic plasticity and neurotransmission,^12,13^ while an alteration of brain cholesterol homeostasis has been linked to Alzheimer's disease (AD).^14^ While the peripheral and central cholesterol pools are separated by the blood-brain barrier, they might interact via oxysterol molecules which could affect neurodegeneration.^11^ More specifically, peripheral hypercholesterolemia has been linked to elevated levels of the oxysterol 27-hydroxycholesterol (27-OHC) which effluxes to the brain from the periphery.^11^ 27-OHC is part of disrupted brain oxysterol homeostasis in AD and may contribute to neurodegeneration.^11,15^ Hypercholesterolemia in midlife is a risk factor for Alzheimer's disease (AD)^16,17^ and high LDL cholesterol in midlife is recognized as one of the 14 modifiable risk factors which could collectively prevent about 45% of dementia according to the 2024 Lancet standing Commission.^18^

Despite numerous clinical trials and observational cohort studies, the effectiveness of statins in preventing AD or slowing cognitive decline after disease onset remains inconclusive. Reports of mild, reversible short-term cognitive side effects^19,20^ prompted the U.S. Food and Drug Administration to issue a warning on statin labelling in 2012.^21^ Large clinical trials examining the risk of AD in statin users generally reported a null effect,^22–25^ or an adverse effect^26^ while more recent trials reported a protective cognitive effect in selected functional domains.^27^ A large number of observational studies, systematic reviews and meta-analyses have generally shown a null^28–30^ or a protective effect.^31–35^ In patients with established AD, clinical trials were probably underpowered and showed a null effect^36,37^ or a beneficial effect.^38,39^ Some observational studies as well as meta-analyses which examined cognitive decline in established AD did not show a meaningful effect of statin treatment.^40–42^

More recent findings from our group have shown a possible cognitive benefit in patients with AD receiving statins.^43^ Studies in animal models reported beneficial effects of lipophilic but possibly low blood-brain barrier-penetrating atorvastatin^9^ which protected against amyloid beta induced hippocampal cell damage in a mouse model^44^ and improved spatial cognition.^45^ Additionally, apolipoprotein E ε4 carriers, who have higher peripheral cholesterol and an elevated risk of AD, may also experience cognitive benefits with statin use.^46^ The *APOE* ε4 allele is associated with a significantly increased risk of AD,^47–49^ and higher LDL-C levels,^50^ while *APOE* ε2 is considered protective for dementia.^51^ One ε4 allele increases AD risk 3–4-fold and lowers the age of onset of dementia.^52,53^ The cognitive effects of statins are complex, with evidence suggesting both beneficial and harmful impacts.^54^ Research on brain and animal models, as well as in AD patients, highlights statins’ anti-inflammatory, antioxidant, and neuroprotective actions.^54^ However, statins may also impair neuronal plasticity and survival.^54^ Negative effects on cognition could stem from mitochondrial dysfunction, increased oxidative stress due to reduced coenzyme Q10, and inhibited cholesterol synthesis, which affects myelination and synaptogenesis.^13^ Statins’ overall impact on cognition likely depends on factors such as pharmacokinetics (potency, blood-brain barrier permeability), length of statin treatment (short- and long-term effects), patient age, comorbidities, genetics, and underlying dementia pathogenesis.^13,55–57^

There is a lack of information on the use and prescription of statins in patients older than 75 years, who often have comorbidities, polypharmacy and have the highest prevalence of Alzheimer's and mixed dementia. These patients are underrepresented in clinical trials.^58,59^ Current recommendations based on international guidelines advise considering statin therapy for primary prevention in older adults, based on individualized assessment of risk factors and potential benefits versus risks.^58,60,61^ Statin therapy is recommended for adults with established cardiovascular disease, regardless of age.^58,61^ The guidelines emphasize shared decision-making between clinicians and patients, considering individual preferences, comorbidities, and life expectancy.

This study aims to understand statin use and its association with cognition at the time of dementia diagnosis. The aims are 1) to determine which factors predict statin use, and specifically use of simvastatin or atorvastatin, and 2) to explore the association between statin use, peripheral cholesterol and cognition at the time of dementia diagnosis. We hypothesized that younger patients and patients with established cardiovascular disease are more likely to receive statins, adhering to guidelines of statin prescription. Moreover, we hypothesized that users of the lipophilic simvastatin which readily crosses the blood-brain barrier^9^ would have better cognitive scores at the time of dementia diagnosis.

## Methods

### Study design and databases

**Figure 1.**
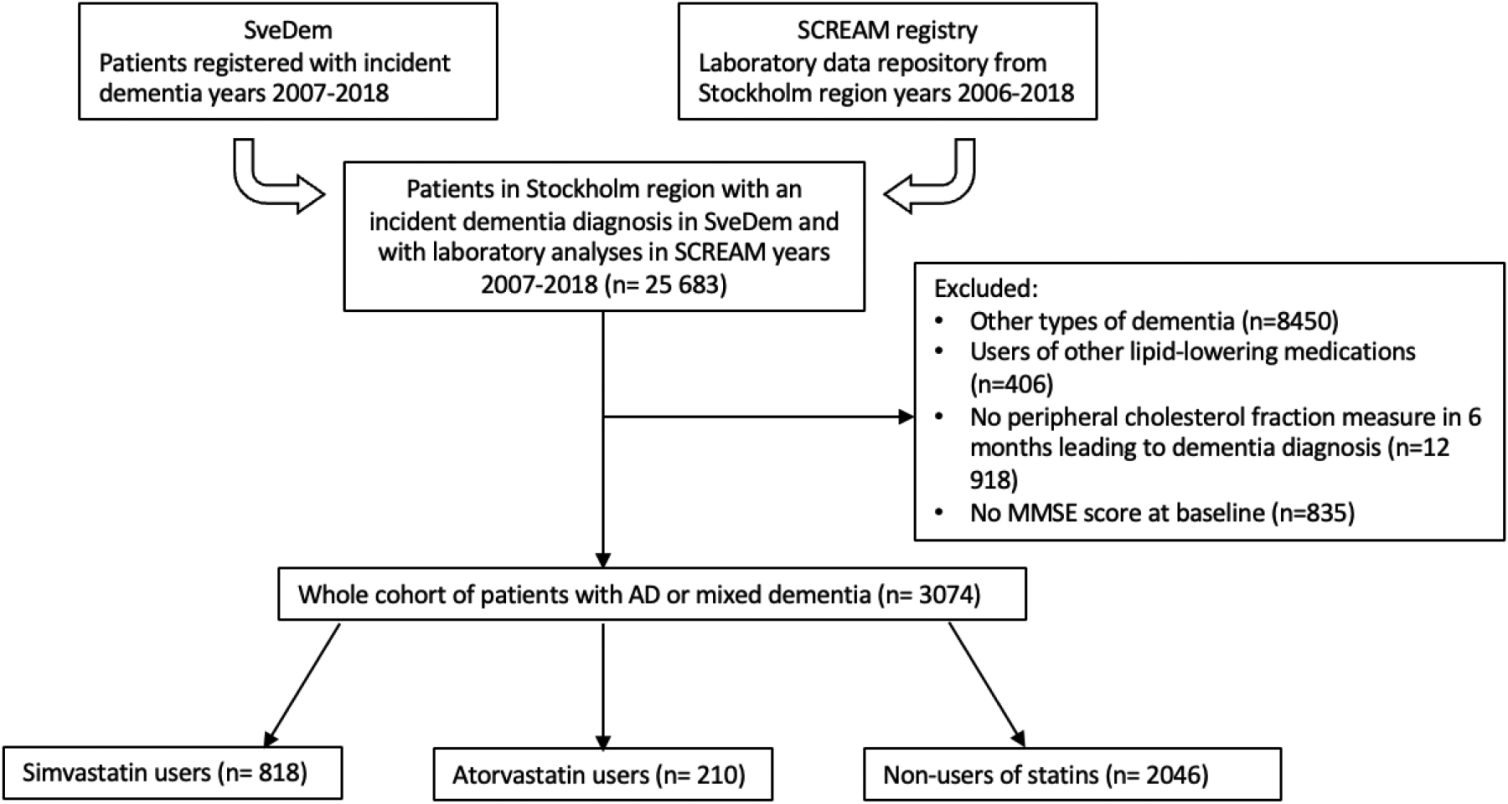
Patient selection flowchart. Our study included patients in the Stockholm region who were registered in SveDem for Alzheimer's disease or mixed dementia from 2007–2018 and had laboratory data available from SCREAM project. We excluded patients with other dementia types, those who did not have MMSE score at diagnosis, patients without cholesterol measures 6 months before dementia diagnosis or patients who used other lipid-lowering medications. Our final cohort consisted of patients with AD or mixed dementia who received simvastatin (n = 818), atorvastatin (n = 210) or did not use statins (n = 2046). SveDem: Swedish Registry for Cognitive/Dementia Disorders; SCREAM: Stockholm Creatinine Measurements Project; MMSE: Mini-Mental State Examination; AD: Alzheimer's disease.

We conducted a cross-sectional study based on Swedish registries including patients with incident Alzheimer's or mixed dementia (ICD-10 codes F001, G301, F002, and G308) from the Swedish Registry for Dementia and Cognitive Disorders (SveDem) and laboratory measures from the Stockholm Creatinine Measurements (SCREAM) project from 01.01.2007 to 31.12.2018.^62,63^ The SCREAM cohort was primarily established to estimate the burden of chronic kidney disease in the region of Stockholm, which houses approximately a quarter of Sweden's total population.^63^ SCREAM links healthcare records and includes the complete population residing in the region, with laboratory data available for 67% of adults^62^ and more than 90% of residents over 65 years.^63^ It also contains comorbidites from primary care, specialist visits and hospitalizations. This registry has previously been linked with other national quality registries utilizing the unique Swedish personal identification number. SveDem is a quality registry, established in 2007 with an aim to improve and monitor the quality of care of patients with dementia in Sweden.^64^ From SveDem, we obtained information on type of dementia according to the ICD-10 codes and cognitive evaluation with Mini-Mental State Examination (MMSE) score at diagnosis. From the Swedish Prescribed Drug Registry which includes all dispensation of prescription medications at pharmacies since July 2005,^65^ we obtained information on medication use according to their ATC codes. In Sweden, statin prescribing is guided both by international recommendations (such as the ESC/EAS Guidelines for the Management of Dyslipidaemias^66,67^) and by national guidelines and treatment recommendations issued by the National Board of Health and Welfare (Socialstyrelsen) and the Medical Products Agency (Läkemedelsverket). The Longitudinal Integration Database for Health Insurance and Labour Market Studies^68^ contributed data on educational levels. The Swedish National Patient Register contains records on hospitalization diagnoses from 1987 and specialized outpatient care from 2001 onwards.^69^ Finally, we used data on date and cause of death from the Swedish Cause of Death Register (Figure 1).

### Statistical analysis

Statin use was defined as a statin prescription within 6 months before dementia diagnosis. The primary outcomes were 1. Statin use and 2. MMSE score at the time of dementia diagnosis. We included age, sex, education level, year of diagnosis, type of diagnostic unit, coresident status, comorbidities, and comedication as covariates in adjusted models (Supplemental Table 1). Information on peripheral cholesterol levels included measurements of total cholesterol (TC), triglycerides (TG), LDL-C and HDL-C within 6 months before dementia diagnosis but after at least one prescription of simvastatin or atorvastatin. We focused on simvastatin and atorvastatin due to their differing degrees of brain penetration and their extensive evaluation in both preclinical and clinical studies on neurodegeneration. Additionally, they were the most prescribed statins in the Swedish registries we analyzed; other statins and other non-statin lipid-lowering medications were used in only 406 patients, making those comparison groups too small for meaningful analysis.

Continuous variables were described with mean (standard deviation, SD) and categorical variables were reported as counts (percentages, %). Odds ratios (OR) with corresponding 95% confidence intervals (CI) were estimated using multivariable logistic regression models to examine the association of baseline characteristics with simvastatin and atorvastatin use. Separate linear regression models were utilized to examine the association of (1) simvastatin or atorvastatin with MMSE score at baseline, and (2) the association of LDL-C, TC, HDL-C, TG levels with MMSE score at baseline, among non-users, users of simvastatin and atorvastatin. Separate models were calculated for LDL-C, TC, HDL-C, and TG. Models were adjusted for age, sex, coresident status, diagnostic unit, educational level, diagnosis year, comorbidities and medications, and missing cases were coded as a separate category and included in the models.

Statistical analyses were performed with SAS, version 9.4 (SAS Institute Inc, Cary, NC) and R 4.2.1 (R Foundation for Statistical Computing). Two-tailed p-values were reported and a value below 0.05 considered significant.

## Results

### Baseline characteristics of study patients

A total of 3074 AD and mixed dementia patients were included in our study. We identified 818 simvastatin users (79.5% of all statin users), 210 atorvastatin users and 2046 non-users of statins. Distribution of defined daily doses (DDD) of simvastatin and atorvastatin is summarized in Supplemental Table 5. The groups did not differ in age at diagnosis (78.4 ± 6.7, 77.7 ± 6.8, 78.1 ± 8.6, respectively), educational level, or type of diagnostic unit. More patients using atorvastatin had been diagnosed in later years. There was a higher proportion of men among simvastatin users (47.3%), compared to atorvastatin (41.4%) or non-users of statins (37.9%). Simvastatin users had a higher MMSE at diagnosis compared to atorvastatin users or non-users of statins (21.7 ± 4.8, 21.6 ± 4.8, 20.9 ± 5.3). More simvastatin users had a coresident (57.7%, 55.7%, 51.1%). Non-users of statins had higher TC, LDL-C, HDL-C and lower TG. Moreover, a higher proportion of patients who used statins had cardiovascular comorbidities and used other medications to treat comorbid conditions. Detailed information is presented in Table 1.

**Table 1.** Baseline characteristics of study patients.

Variables	Statin nonuser (n = 2 046)	Simvastatin (n = 818)	Atorvastatin (n = 210)
Age at dementia diagnosis, y			
Mean (SD)	78.1 (8.6)	78.4 (6.7)	77.7 (6.8)
< 65	180 (8.8)	26 (3.2)	10 (4.7)
65–75	431 (21.0)	183 (22.3)	52 (24.2)
75–85	944 (45.9)	466 (56.7)	119 (55.3)
≥ 85	500 (24.3)	147 (17.9)	34 (15.8)
Sex			
Male	775 (37.9)	387 (47.3)	87 (41.4)
Female	1271 (62.1)	431 (52.7)	123 (58.6)
Educational level			
Completed compulsory education	629 (30.7)	251 (30.7)	56 (26.7)
Upper secondary	837 (40.9)	342 (41.8)	98 (46.7)
College/university	523 (25.6)	206 (25.2)	55 (26.2)
Missing	57(2.8)	19(2.3)	1 (0.5)
Coresident status			
Cohabiting	1045 (51.1)	472 (57.7)	117 (55.7)
Living alone	949 (46.4)	330 (40.3)	91 (43.3)
Missing	52 (2.5)	16 (2.0)	2 (1.0)
Type of diagnostic unit			
Specialist care	2036 (99.5)	816 (99.8)	208 (99.0)
Primary care	10 (0.5)	2 (0.2)	2 (1.0)
Calendar year of diagnosis			
2007–2009	292 (14.3)	98 (12.0)	9 (4.3)
2010–2012	543 (26.5)	201 (24.6)	13 (6.2)
2013–2015	587 (28.7)	237 (29.0)	41 (19.5)
2016–2018	624 (30.5)	282 (34.5)	147 (70.0)
MMSE score			
Mean (SD)	20.9 (5.3)	21.7 (4.8)	21.6 (4.8)
0–19	677 (33.1)	232 (28.4)	65 (31.0)
20–24	786 (38.4)	333 (40.7)	72 (34.3)
25–30	583 (28.5)	253 (30.9)	73 (34.8)
Comorbidities			
Hyperlipidemia	22 (1.1)	104 (12.7)	42 (20.0)
Hypertension	527 (25.8)	331 (40.5)	89 (42.4)
Diabetes mellitus	215 (10.5)	238 (29.1)	48 (22.9)
Type 1 diabetes mellitus	24 (1.2)	28 (3.4)	7 (3.3)
Type 2 diabetes mellitus	199 (9.7)	221 (27.0)	43 (20.5)
Atrial fibrillation	242 (11.8)	139 (17.0)	49 (23.3)
Angina pectoris	57 (2.8)	68 (8.3)	21 (10.0)
Congestive heart failure	107 (5.2)	83 (10.1)	20 (9.5)
Myocardial infarction	53 (2.6)	92 (11.2)	43 (20.5)
Stroke	95 (4.6)	96 (11.7)	29 (13.8)
Ischemic heart disease	108 (5.3)	155 (18.9)	57 (27.1)
Chronic respiratory disease	100 (4.9)	51 (6.2)	13 (6.2)
Liver disease	9 (0.4)	3 (0.4)	1 (0.5)
Cancer	314 (15.3)	133 (16.3)	41 (19.5)
Fracture	246 (12.0)	99 (12.1)	18 (8.6)
Chronic kidney disease	502 (24.5)	246 (30.1)	55 (26.2)
Alcohol-related diseases	43 (2.1)	15 (1.8)	1 (0.5)
Depression	114 (5.6)	50 (6.1)	10 (4.8)
Medication use			
Cardiac drugs	367 (17.9)	270 (33.0)	75 (35.7)
Antihypertensives	45 (2.2)	26 (3.2)	10 (4.8)
Diuretics	448 (21.9)	268 (32.8)	62 (29.5)
Peripheral vasodilators	44 (2.2)	73 (8.9)	27 (12.9)
Vasoprotective drugs	121 (5.9)	50 (6.1)	26 (12.4)
β-blocking agents	528 (25.8)	451 (55.1)	118 (56.2)
Calcium channel blockers	387 (18.9)	244 (29.8)	62 (29.5)
RAAS	633 (30.9)	475 (58.1)	139 (66.2)
Insulin	86 (4.2)	111 (13.6)	26 (12.4)
Other antidiabetics	154 (7.5)	183 (22.4)	39 (18.6)
NSAIDs	138 (6.7)	57 (7.0)	16 (7.6)
Vitamin D	63 (3.1)	26 (3.2)	13 (6.2)
Anticoagulants	201 (9.8)	144 (17.6)	48 (22.9)
Antiplatelets	482 (23.6)	478 (58.4)	114 (54.3)
Anxiolytics	263 (12.9)	92 (11.2)	26 (12.4)
Hypnotics	365 (17.8)	189 (23.1)	52 (24.8)
Antipsychotics	107 (5.2)	28 (3.4)	7 (3.3)
Antidepressants	435 (21.3)	230 (28.1)	54 (25.7)
TC, mmol/L, Mean (SD)	5.5 (1.1)	4.4 (1.0)	4.6 (1.1)
LDL-C, mmol/L, Mean (SD)	3.3 (0.9)	2.4 (0.9)	2.4 (0.9)
HDL-C, mmol/L, Mean (SD)^a^	1.7 (0.5)	1.5 (0.5)	1.6 (0.5)
TG, mmol/L, Mean (SD)^b^	1.2 (0.6)	1.3 (0.6)	1.3 (0.7)

SD: standard deviation; MMSE: Mini-Mental State Examination; RAAS: Agents acting on the renin-angiotensin system; TC: total cholesterol; LDL-C: low-density lipoprotein cholesterol; HDL: high-density lipoprotein cholesterol; TG: triglycerides.

^a^ n = 1710 for nonuser, n = 670 for simvastatin, n = 147 for atorvastatin; ^b^n = 1917 for nonuser, n = 780 for simvastatin, n = 206 for atorvastatin.

### Factors associated with the use of statins

Older age was associated with lower odds of receiving either simvastatin (OR 0.98, 95% CI 0.97–0.99) or atorvastatin (OR 0.97, 95% CI 0.94–0.99). Diabetes mellitus (OR 2.25, 95% CI 1.79–2.82), stroke (OR 1.75, 95% CI 1.25–2.45) or ischemic heart disease (OR 2.48, 95% CI 1.83–3.35) were associated with greater odds of statin use, whereas congestive heart failure was associated with lower odds of receiving atorvastatin, compared to no statin (OR 0.47, 95% CI 0.25–0.86) (Table 2 and Supplemental Table 3). Analyses stratified by dementia type (AD versus mixed dementia) showed similar results (not presented).

**Table 2.** Factors associated with the use of simvastatin or atorvastatin compared to non-use of statins.

Variables	Simvastatin (n = 818)		Atorvastatin (n = 210)	
	OR (95%CI)	*p*	OR (95%CI)	*p*
Age at dementia diagnosis, y	0.98 (0.97–0.99)	0.005	0.97 (0.94–0.99)	0.002
Sex				
Male	REF		REF	
Female	0.84 (0.69–1.02)	0.076	1.14 (0.82–1.58)	0.434
Educational level				
Completed compulsory education	REF		REF	
Upper secondary	0.86 (0.46–1.58)	0.618	0.19 (0.03–1.48)	0.113
College/university	1.21 (0.94–1.55)	0.144	1.44 (0.94–2.19)	0.092
Missing	1.12 (0.90–1.40)	0.303	1.38 (0.96–2.00)	0.087
Coresident status				
Cohabiting	REF		REF	
Living alone	0.72 (0.38–1.38)	0.328	0.32 (0.07–1.44)	0.137
Missing	0.75 (0.61–0.91)	0.004	0.83 (0.60–1.15)	0.256
Comorbidities				
Diabetes mellitus	2.39 (1.89–3.02)	<0.001	1.73 (1.18–2.54)	0.005
Atrial fibrillation	0.89 (0.67–1.19)	0.442	1.51 (0.98–2.33)	0.064
Congestive heart failure	0.70 (0.47–1.03)	0.068	0.47 (0.25–0.86)	0.014
Stroke	1.71 (1.20–2.42)	0.003	1.98 (1.19–3.29)	0.009
Ischemic heart disease	2.17 (1.58–2.99)	<0.001	3.98 (2.57–6.15)	<0.001
Chronic kidney disease	0.94 (0.75–1.17)	0.578	0.81 (0.56–1.17)	0.264
Medication use				
Diuretics	1.18 (0.95–1.47)	0.144	0.96 (0.67–1.38)	0.832
Peripheral vasodilators	1.93 (1.24–3.00)	0.004	2.74 (1.53–4.91)	0.001
Vasoprotective drugs	0.90 (0.62–1.32)	0.590	2.16 (1.33–3.52)	0.002
β-blocking agents	2.29 (1.86–2.81)	<0.001	1.97 (1.40–2.78)	<0.001
Calcium channel blockers	1.26 (1.02–1.56)	0.033	1.20 (0.85–1.68)	0.305
RAAS	2.09 (1.73–2.54)	<0.001	3.38 (2.44–4.68)	<0.001
Antiplatelets	2.83 (2.32–3.44)	<0.001	2.38 (1.70–3.34)	<0.001
Hypnotics	1.32 (1.04–1.66)	0.022	1.46 (1.00–2.12)	0.048
Antipsychotics	0.54 (0.33–0.87)	0.012	0.47 (0.20–1.11)	0.085
Antidepressants	1.25 (1.00–1.55)	0.047	0.97 (0.68–1.40)	0.884

RAAS: Agents acting on the renin-angiotensin system.

Simvastatin users had 0.53 points higher MMSE at baseline, compared to non-users of statins (se 0.23, p = 0.021, adjusted model). Analysis stratified by sex showed 0.73 points higher MMSE in male simvastatin users (se 0.36, p = 0.042, adjusted model) whereas female simvastatin users had 0.34 higher MMSE points, which was not statistically significant (p = 0.261) (Table 3).

**Table 3.** Association between statin use and MMSE score at diagnosis.

Variables	Crude model		Adjusted model		Male, adjusted model^a^		Female, adjusted model^b^	
	β (se)	*p*	β (se)	*p*	β (se)	*p*	β (se)	*p*
Nonuser (n = 2 046)	REF		REF		REF		REF	
Simvastatin (n = 818)	0.72 (0.21)	0.007	0.53 (0.23)	0.021	0.73 (0.36)	0.042	0.34 (0.30)	0.261
Atorvastatin (n = 210)	0.67 (0.37)	0.073	0.27 (0.38)	0.475	0.27 (0.61)	0.665	0.31 (0.49)	0.528

Model adjusted for age, coresident status, diagnostic unit, educational level, diagnosis year, comorbidities and medications. MMSE: Mini-Mental State Examination.

^a^ n = 775 for nonuser, n = 387 for simvastatin, n = 87 for atorvastatin; ^b^n = 1271 for nonuser, n = 431 for simvastatin, n = 123 for atorvastatin.

Higher LDL-C, TC and HDL-C levels at baseline were associated with higher MMSE in non-users of statins in separate adjusted models (β 0.32 (se 0.13, p = 0.013), 0.47 (se 0.11, p < 0.001), 1.02 (se 0.26, p < 0.001), respectively). The association had a very small effect size and was not statistically significant for simvastatin or atorvastatin users. (Tables 4 and 5). The interaction terms between sex and statin use or sex and cholesterol on MMSE score was not significant.

**Table 4.** The association between LDL-C, TC, HDL-C, TG and MMSE score at diagnosis among non-users, and users of simvastatin and atorvastatin.

Variables	Nonuser		Simvastatin		Atorvastatin	
	β (se)	*p*	β (se)	*p*	β (se)	*p*
Crude model						
LDL-C (n = 2046)	0.43 (0.13)	0.001	0.40 (0.19)	0.038	0.33 (0.35)	0.353
TC (n = 2046)	0.55 (0.11)	<0.001	0.30 (0.16)	0.065	0.34 (0.30)	0.259
TG (n = 1917)^a^	0.06 (0.20)	0.776	0.39 (0.27)	0.159	0.58 (0.49)	0.234
HDL-C (n = 1710)^b^	1.09 (0.24)	<0.001	−0.48 (0.39)	0.219	−0.20 (0.80)	0.798
Adjusted model						
LDL-C (n = 2046)	0.32 (0.13)	0.013	0.11 (0.20)	0.572	0.22 (0.36)	0.545
TC (n = 2046)	0.47 (0.11)	<0.001	0.11 (0.18)	0.518	0.19 (0.31)	0.555
TG (n = 1917)^a^	0.06 (0.20)	0.766	0.38 (0.28)	0.170	0.83 (0.47)	0.081
HDL-C (n = 1710)^b^	1.02 (0.26)	<0.001	−0.30 (0.41)	0.472	−0.25 (0.86)	0.767

^a^ n = 1917 for nonuser, n = 780 for simvastatin, n = 206 for atorvastatin; ^b^n = 1710 for nonuser, n = 670 for simvastatin, n = 147 for atorvastatin.

Model adjusted for age, sex, education level, coresident status, type of diagnostic unit, calendar year of dementia diagnosis, comorbidities and medications.

**Table 5.** The association between LDL-C, TC, HDL-C, TG and MMSE score at diagnosis among non-users, and users of simvastatin and atorvastatin, stratified by sex.

Variables	Nonuser			Simvastatin			Atorvastatin		
	n	β (se)	*p*	n	β (se)	*p*	n	β (se)	*p*
Men									
LDL-C	775	0.05 (0.24)	0.837	387	−0.34 (0.32)	0.29	87	0.03 (0.55)	0.960
TC	775	0.19 (0.21)	0.381	387	−0.26 (0.28)	0.357	87	0.14 (0.50)	0.781
TG	734	−0.14 (0.32)	0.670	372	0.06 (0.41)	0.889	86	0.11 (0.69)	0.875
HDL-C	643	1.23 (0.49)	0.013	317	−0.92 (0.64)	0.151	61	0.56 (1.72)	0.747
Women									
LDL-C	1271	0.39 (0.15)	0.011	431	0.32 (0.26)	0.204	123	0.39 (0.51)	0.448
TC	1271	0.53 (0.14)	<.001	431	0.31 (0.23)	0.176	123	0.14 (0.43)	0.739
TG	1183	0.16 (0.26)	0.532	408	0.65 (0.37)	0.081	120	1.56 (0.68)	0.024
HDL-C	1067	0.82 (0.31)	0.009	353	0.12 (0.54)	0.832	86	−0.75 (1.13)	0.509

Model adjusted for age, education level, coresident status, type of diagnostic unit, calendar year of dementia diagnosis, comorbidities and medications.

## Discussion

In our nationwide Swedish registry study including patients with AD or mixed dementia, we observed the following key findings: (1) Older patients were less likely to receive simvastatin or atorvastatin, (2) simvastatin users had a better cognition (measured as MMSE score) at the time of diagnosis, compared to non-users of statins, (3) higher LDL-C, TC and HDL-C levels were associated with a better MMSE at baseline in non-users of statins.

In Sweden, simvastatin was prescribed more frequently according to national guidelines considering the cost and benefit, but this has changed in later years, following the international guidelines that advise moderate to high-intensity statin use in cardiovascular disease prevention.^60,61,70^ A shift to prescribing atorvastatin from simvastatin has been observed in Sweden in the later years.^71^ In our cohort 80% of statin users were taking simvastatin. Patients with ischemic heart disease were more likely to receive statins and were almost four times more likely to receive atorvastatin, reflecting the preference of this statin in patients with atherosclerotic disease. On the other hand, older patients were less likely to be prescribed statins in our study. For every one-year increase in age at dementia diagnosis, the adjusted likelihood of receiving either simvastatin or atorvastatin at baseline decreased by approximately 2%. These results may reflect the decision of prescribing statins in older individuals, based on the balance between the cardiovascular benefits, potential risk for adverse effects, and estimated survival. A large general population study including over 7 million participants in UK reported growing initiation and growing prevalent use of statins in older patients which declined only after age 85.^1^ Another study found that people aged 65 to 75 were more likely to receive statins than those younger or older, and high-intensity statins were prescribed more frequently to people 65 and younger, and least frequently to those 75 or older.^3^

In our study, simvastatin users had 0.53 points higher MMSE on average at dementia diagnosis, compared to non-users of statins. Analysis stratified on sex showed the association was driven by men who had 0.73 higher MMSE points compared to male simvastatin non-users, while the association was not significant in women. In our previous study including 15,586 Alzheimer's and mixed dementia patients, we observed a dose-dependent, slower cognitive decline over time in simvastatin users compared to non-users of statins.^43^ In the early stages of AD, certain patients may experience disrupted central lipid metabolism and elevated neuroinflammation levels, and treatment with simvastatin could theoretically improve these issues.^54^ Simvastatin's ability to cross the blood-brain barrier allows it to exert various neuroprotective actions, including protection against tau phosphorylation,^9^ anti-inflammatory effects, promotion of hippocampal neurogenesis as well as enhancement of neurotropic factors.^72^ Moreover, atorvastatin demonstrated beneficial cognitive effects in mouse models, which included an improved spatial cognition^45^ as well as reversal of hippocampal cell damage by Aβ_40_.^44^ However, despite being lipophilic, its brain penetration may be limited due to its large molecular size.^9^ Clinical trials involving patients with mild to moderate AD have reported either null effects of simvastatin^36^ or atorvastatin^37^ or a potential cognitive benefits from simvastatin^39^ or atorvastatin treatment^38^ with MMSE as an outcome. Additionally, sex-related differences in pharmacokinetics and pharmacodynamics of lipid metabolism statins have been a longstanding topic of debate, influenced by various factors, including hormonal status and its impact on cardiovascular risk profiles.^73–75^ Cholesterol serves as the substrate for sex hormone biosynthesis suggesting an influence of statins on sex hormones levels.^76^ However, the overall role of testosterone in the brain in cognitive functions is still not clear.^77,78^ Most studies did not find a significant effect of statins on serum testosterone levels,^79^ though simvastatin has been linked to dose- and duration-dependent reductions.^80,81^ On the other hand, faster statin metabolism in women, due to higher Cytochrome P450 3A4 activity, may reduce the effectiveness of lipophilic statins like atorvastatin and simvastatin.^74,75^ Moreover, we must consider possible epidemiological biases inherent to the design of our study. Notably, we did not differentiate between new and prevalent users of statins due to the small number of new users in this cohort, and this could lead to prevalent-user bias, limiting our findings.

Additionally, higher levels of LDL-C, total cholesterol and HDL-C were associated with better cognitive scores at dementia diagnosis in non-users of statins in our study. While hypercholesterolemia in midlife has been recognized as one of the risk factors for AD in late life,^16^ higher levels of cholesterol in late life have been connected to a reduced risk of dementia^82,83^ and reflect less frailty and better overall health.^84^ High LDL cholesterol may be associated with better cognitive scores in non-users of statins because it could reflect better overall health, non-AD pathologies, nutritional status, or cholesterol's role in brain function in that group. In contrast, statin use by lowering cholesterol without impacting nutrition or frailty, may disrupt the statistical association between cholesterol and cognition.

An interesting study including more than 450,000 participants from the UK biobank cohort investigated cross-sectional and longitudinal associations between statin use and cognitive performance in patients aged 40–69 without dementia.^55^ In this study, statin use was associated with negative effects on cognition at baseline through lowering LDL-C (proportion mediated 51.4%). However, after 8 years of follow-up, there was no association between statin use and cognition which may suggest that the long-term use of statins could outweigh these short-term adverse effects by diminishing cardiovascular risk which promotes AD. Moreover, the relationship between LDL-C levels and cognition may be U-shaped as shown in previous research.^85^ A Mendelian randomization study by Williams et al. did not find support for a preventive effect on AD of lowering LDL-C by pathways related to statins, ezetimibe, or PCSK9 inhibitors.^86^ In line with our results, this suggests that the rationale for repurposing statins in the cognitive field may be based on mechanisms other than LDL-C lowering. Such pleiotropic mechanisms may include mitigation of inflammation, antioxidative effects or transcriptional effects on genes involved in processes such as cell growth, structure and signaling as well as glucose metabolism.^10,14,55^ The variability in statin response, lipid profile variations as well as genetic factors in relation to AD has been further elucidated through pharmacogenetic studies. De Oliveira et al. demonstrated that selected protective variants of *LDLR* and *APOE* were associated with reduced risk of late-onset AD and slower cognitive decline, regardless of cholesterol variations, and that carriers of specific genetic variants benefited from lipophilic statins.^87^ In another study, these authors have reported that selected protective variants of *CETP* and *NR1H2* against risk of AD also slowed cognitive and functional decline for *APOE* ε4 carriers in particular, regardless of cholesterol variations, while receiving lipophilic statins might affect carriers of specific genetic variants.^88^ Moreover, *APOE* haplotypes appears to modulate the effects of lipid profile changes in AD dementia.^89^ APOE4- haplotypes may enhance lipid availability to support neuronal membrane integrity with rising cholesterol levels, whereas *APOE* ε4 carriers did not show any cognitive effects of lipid profile variations as these patients exhibit less efficient neural repair. In this study, lipophilic statins had non-significant protective effects for *APOE4*- carriers only.^89^ In conclusion, these studies allow for a more personalized understanding of lipid profile variations and genetic factors related to cognition as well as the effects of statins on cognitive function in AD patients.^87–89^

The selection of our cohort through combined large national quality Swedish registries is an important strength of our study. However, a few limitations must be noted. Cross-sectional designs can be biased by unmeasured confounding and possible reverse causation, e.g., patients with a better cognition having a higher likelihood of receiving the statins. Moreover, it does not allow to draw any conclusions on cognitive trajectory. Basal concentrations of LDL-C affect the likelihood of receiving statins, which in turn has a treatment-feedback effect on LDL-C. This might have contributed to bias and should be considered in future longitudinal studies. Another limitation is that cholesterol levels were assessed at a single time point within six months prior to diagnosis, which may not reliably reflect long-term lipid profiles. This is particularly relevant in older adults or individuals with advanced disease, where cholesterol levels are more prone to fluctuation. We indirectly assumed patients adhered to medications they obtained from the pharmacies. Moreover, information on duration of statin therapy were not included in our study. Since the medication registry was established in 2005, it is unfortunately not possible to determine from our data whether most patients in our cohort initiated statin therapy during midlife or later. Importantly, the registries we used for our study do not include genetic factors, such as *APOE* haplotypes, or other relevant genetic polymorphisms relevant to the metabolism or transport of statins. Moreover, we did not have the information regarding dietary therapy for these patients. Additionally, a half-point difference on the 30-point MMSE may not be clinically relevant, although it could become relevant over follow-up if effects confound, which we will investigate in future studies. In the future, longitudinal analyses including these factors are warranted to determine the long-term cognitive effects of statins by their cholesterol-lowering effects as well as other potential pathways for further stratification and personalized medicine intervention strategies for AD.

### Conclusions

In this study, younger age and cardiovascular comorbidities were associated with higher likelihood of statin prescription. Statin use was associated with higher MMSE scores at the time of dementia diagnosis. In non-users, higher LDL-C, total cholesterol, and HDL-C levels correlated with better baseline cognitive scores. Although limited by cross-sectional design, this study provides valuable insights for designing future longitudinal investigations of statins and cognition. Baseline MMSE is a strong predictor of cognitive decline, and pre-existing differences between treated and untreated patients must be considered when interpreting cognitive trajectories. Additionally, factors influencing statin prescription, as outlined in this paper, are critical both for optimizing treatment and access in dementia populations and for addressing potential confounding in longitudinal cognitive research.

## Supplemental Material

sj-docx-1-alr-10.1177_25424823251385903 - Supplemental material for Statins, cholesterol and cognition at the time of Alzheimer's disease diagnosis: A cross-sectional study from the Swedish registry for cognitive/dementia disorders Supplemental material, sj-docx-1-alr-10.1177_25424823251385903 for Statins, cholesterol and cognition at the time of Alzheimer's disease diagnosis: A cross-sectional study from the Swedish registry for cognitive/dementia disorders by Bojana Petek, Minjia Mo, Hong Xu, Jakob Norgren, Minh Tuan Hoang, Marta Villa-Lopez, Henrike Häbel, Julianna Kele, Luana Naia, Silvia Maioli, Joana B Pereira, Milica Gregorič Kramberger, Bengt Winblad, Maria Eriksdotter, Juan-Jesus Carrero and Sara Garcia-Ptacek in Journal of Alzheimer's Disease Reports

## References

[1] O’Keeffe AG Nazareth I Petersen I . Time trends in the prescription of statins for the primary prevention of cardiovascular disease in the United Kingdom: a cohort study using the health improvement network primary care data. Clin Epidemiol 2016; 8: 123–132. 27313477 10.2147/CLEP.S104258 PMC4890684

[2] Svensson E Nielsen RB Hasvold P , et al. Statin prescription patterns, adherence, and attainment of cholesterol treatment goals in routine clinical care: a Danish population-based study. Clin Epidemiol 2015; 7: 213–223. 25759601 10.2147/CLEP.S78145 PMC4345937

[3] Yao X Shah ND Gersh BJ , et al. Assessment of trends in statin therapy for secondary prevention of atherosclerotic cardiovascular disease in US adults from 2007 to 2016. JAMA Netw Open 2020; 3: e202117. 10.1001/jamanetworkopen.2020.25505 PMC7679951 33216139

[4] Sirtori CR . The pharmacology of statins. Pharmacol Res 2014; 88: 3–11. 24657242 10.1016/j.phrs.2014.03.002

[5] Nicholls SJ Tuzcu EM Sipahi I , et al. Statins, high-density lipoprotein cholesterol, and regression of coronary atherosclerosis. JAMA 2007; 297: 499–508. 17284700 10.1001/jama.297.5.499

[6] Illingworth DR Crouse JR Hunninghake DB , et al. A comparison of simvastatin and atorvastatin up to maximal recommended doses in a large multicenter randomized clinical trial. Curr Med Res Opin 2001; 17: 43–50. 11464446

[7] Oh RC Trivette ET Westerfield KL . Management of hypertriglyceridemia: common questions and answers. Am Fam Physician 2020; 102: 347–354. 32931217

[8] McFarland AJ Anoopkumar-Dukie S Arora DS , et al. Molecular mechanisms underlying the effects of statins in the central nervous system. Int J Mol Sci 2014; 15: 20607–20637. 25391045 10.3390/ijms151120607 PMC4264186

[9] Sierra S Ramos MC Molina P , et al. Statins as neuroprotectants: a comparative in vitro study of lipophilicity, blood–brain barrier penetration, lowering of brain cholesterol, and decrease of neuron cell death. J Alzheimers Dis 2011; 23: 307–318. 21098985 10.3233/JAD-2010-101179

[10] Petek B Villa-Lopez M Loera-Valencia R , et al. Connecting the brain cholesterol and renin–angiotensin systems: potential role of statins and RAS-modifying medications in dementia. J Intern Med 2018; 284: 620–642. 30264910 10.1111/joim.12838

[11] Loera-Valencia R Goikolea J Parrado-Fernandez C , et al. Alterations in cholesterol metabolism as a risk factor for developing Alzheimer’s disease: potential novel targets for treatment. J Steroid Biochem Mol Biol 2019; 190: 104–114. 30878503 10.1016/j.jsbmb.2019.03.003

[12] Chew H Solomon VA Fonteh AN . Involvement of lipids in Alzheimer’s disease pathology and potential therapies. Front Physiol 2020; 11: 598. 32581851 10.3389/fphys.2020.00598 PMC7296164

[13] Tan B Rosenfeldt F Ou R , et al. Evidence and mechanisms for statin-induced cognitive decline. Expert Rev Clin Pharmacol 2019; 12: 397–406. 31030614 10.1080/17512433.2019.1606711

[14] Sodero AO Barrantes FJ . Pleiotropic effects of statins on brain cells. Biochim Biophys Acta Biomembr 2020; 1862: 183223. 10.1016/j.bbamem.2020.183340 32387399

[15] Björkhem I Cedazo-Minguez A Leoni V , et al. Oxysterols and neurodegenerative diseases. Mol Aspects Med 2009; 30: 171–179. 19248803 10.1016/j.mam.2009.02.001

[16] Kivipelto M Solomon A . Cholesterol as a risk factor for Alzheimer’s disease: epidemiological evidence. Acta Neurol Scand 2006; 114: 50–57. 10.1111/j.1600-0404.2006.00685.x 16866911

[17] Solomon A Kåreholt I Ngandu T , et al. Serum cholesterol changes after midlife and late-life cognition: 21-year follow-up study. Neurology 2007; 68: 751–756. 17339582 10.1212/01.wnl.0000256368.57375.b7

[18] Livingston G Huntley J Liu KY , et al. Dementia prevention, intervention, and care: 2024 report of the Lancet standing commission. Lancet 2024; 404: 572–628. 39096926 10.1016/S0140-6736(24)01296-0

[19] Evans MA Golomb BA . Statin-associated adverse cognitive effects: survey results from 171 patients. Pharmacotherapy 2009; 29: 800–811. 19558254 10.1592/phco.29.7.800

[20] Wagstaff LR Mitton MW Arvik BML , et al. Statin-associated memory loss: analysis of 60 case reports and review of the literature. Pharmacotherapy 2003; 23: 871–880. 12885101 10.1592/phco.23.7.871.32720

[21] US Food and Drug Administration . FDA Drug Safety Communication: important safety label changes to cholesterol-lowering statin drugs. [Internet]. 2012. Available at: https://www.fda.gov/drugs/drug-safety-and-availability/fda-drug-safety-communication-important-safety-label-changes-cholesterol-lowering-statin-drugs (accessed 9 December 2024).

[22] Collins R Armitage J Parish S , et al. MRC/BHF heart protection study of cholesterol lowering with simvastatin in 20,536 high-risk individuals: a randomised placebo-controlled trial. Lancet 2002; 360: 7–22. 12114036 10.1016/S0140-6736(02)09327-3

[23] Trompet S van Vliet P de Craen AJM , et al. Pravastatin and cognitive function in the elderly: results of the PROSPER study. J Neurol 2010; 257: 85–90. 19653027 10.1007/s00415-009-5271-7

[24] Summers MJ Oliver KR Coombes JS , et al. Effect of atorvastatin on cognitive function in patients from the lipid lowering and onset of renal disease (LORD) trial. Pharmacotherapy 2007; 27: 183–190. 17253908 10.1592/phco.27.2.183

[25] Bosch J O’Donnell M Swaminathan B , et al. Effects of blood pressure and lipid lowering on cognition: results from the HOPE-3 study. Neurology 2019; 92: e1435–e1446. 10.1212/WNL.0000000000007174 PMC6453765 30814321

[26] Muldoon MF Ryan CM Sereika SM , et al. Randomized trial of the effects of simvastatin on cognitive functioning in hypercholesterolemic adults. Am J Med 2004; 117: 823–829. 15589485 10.1016/j.amjmed.2004.07.041

[27] Bosch JJ O’Donnell MJ Gao P , et al. Effects of a polypill, aspirin, and the combination of both on cognitive and functional outcomes. JAMA Neurol 2023; 80: 251–259. 36716007 10.1001/jamaneurol.2022.5088 PMC9887530

[28] Samaras K Brodaty H Sachdev PS . Does statin use cause memory decline in the elderly? Trends Cardiovasc Med 2016; 26: 550–565. 27177529 10.1016/j.tcm.2016.03.009

[29] McGuinness B Craig D Bullock R , et al. Statins for the prevention of dementia. Cochrane Database Syst Rev 2016; 2016: CD003160. 10.1002/14651858.CD003160.pub3 PMC9346344 26727124

[30] Adhikari A Tripathy S Chuzi S , et al. Association between statin use and cognitive function: a systematic review of randomized clinical trials and observational studies. J Clin Lipidol 2021; 15: 22–32.e12. 33189626 10.1016/j.jacl.2020.10.007

[31] Richardson K Schoen M French B , et al. Statins and cognitive function: a systematic review. Ann Intern Med 2013; 159: 688–697. 24247674 10.7326/0003-4819-159-10-201311190-00007

[32] Chu CS Tseng PT Stubbs B , et al. Use of statins and the risk of dementia and mild cognitive impairment: a systematic review and meta-analysis. Sci Rep 2018; 8: 5804. 29643479 10.1038/s41598-018-24248-8 PMC5895617

[33] Zhu XC Dai WZ Ma T . Overview of the effect of statin therapy on dementia risk, cognitive changes and its pathologic change: a systematic review and meta-analysis. Ann Transl Med 2018; 6: 435. 30596065 10.21037/atm.2018.06.43 PMC6281522

[34] Poly TN Islam MM Walther BA , et al. Association between use of statin and risk of dementia: a meta-analysis of observational studies. Neuroepidemiology 2020; 54: 214–226. 31574510 10.1159/000503105

[35] Olmastroni E Molari G De Beni N , et al. Statin use and risk of dementia or Alzheimer’s disease: a systematic review and meta-analysis of observational studies. Eur J Prev Cardiol 2022; 29: 804–814. 34871380 10.1093/eurjpc/zwab208

[36] Sano M Bell KL Galasko D , et al. A randomized, double-blind, placebo-controlled trial of simvastatin to treat Alzheimer disease. Neurology 2011; 77: 556–563. 21795660 10.1212/WNL.0b013e318228bf11 PMC3149154

[37] Feldman HH Doody RS Kivipelto M , et al. Randomized controlled trial of atorvastatin in mild to moderate Alzheimer disease: LEADe. Neurology 2010; 74: 956–964. 20200346 10.1212/WNL.0b013e3181d6476a

[38] Sparks DL Sabbagh MN Connor DJ , et al. Atorvastatin for the treatment of mild to moderate Alzheimer disease: preliminary results. Arch Neurol 2005; 62: 753–757. 15883262 10.1001/archneur.62.5.753

[39] Simons M Schwärzler F Lütjohann D , et al. Treatment with simvastatin in normocholesterolemic patients with Alzheimer’s disease: a 26-week randomized, placebo-controlled, double-blind trial. Ann Neurol 2002; 52: 346–350. 12205648 10.1002/ana.10292

[40] Murphy C Dyer AH Lawlor B , et al. What is the impact of ongoing statin use on cognitive decline and dementia progression in older adults with mild–moderate Alzheimer disease? PLoS One 2023; 18: e0285634. 10.1371/journal.pone.0285529 PMC10174559 37167234

[41] Liang T Li R Cheng O . Statins for treating Alzheimer’s disease: truly ineffective? Eur Neurol 2015; 73: 360–366. 26021802 10.1159/000382128

[42] Davis KAS Bishara D Perera G , et al. Benefits and harms of statins in people with dementia: a systematic review and meta-analysis. J Am Geriatr Soc 2020; 68: 650–658. 32039479 10.1111/jgs.16342

[43] Petek B Häbel H Xu H , et al. Statins and cognitive decline in patients with Alzheimer’s and mixed dementia: a longitudinal registry-based cohort study. Alzheimers Res Ther 2023; 15: 142. 38115091 10.1186/s13195-023-01360-0 PMC10731754

[44] Ludka FK Cunha MP Dal-Cim T , et al. Atorvastatin protects from Aβ1-40-induced cell damage and depressive-like behavior via proBDNF cleavage. Mol Neurobiol 2017; 54: 6163–6173. 27709490 10.1007/s12035-016-0134-6

[45] Zhao L Chen T Wang C , et al. Atorvastatin in improvement of cognitive impairments caused by amyloid β in mice: involvement of inflammatory reaction. BMC Neurol 2016; 16: 175. 26846170 10.1186/s12883-016-0533-3 PMC4743318

[46] Geifman N Brinton RD Kennedy RE , et al. Evidence for benefit of statins to modify cognitive decline and risk in Alzheimer’s disease. Alzheimers Res Ther 2017; 9: 10. 28212683 10.1186/s13195-017-0237-y PMC5316146

[47] Serrano-Pozo A Das S Hyman BT . APOE And Alzheimer’s disease: advances in genetics, pathophysiology, and therapeutic approaches. Lancet Neurol 2021; 20: 68–80. 33340485 10.1016/S1474-4422(20)30412-9 PMC8096522

[48] Huang Y Mahley RW . Apolipoprotein E: structure and function in lipid metabolism, neurobiology, and Alzheimer’s diseases. Neurobiol Dis 2014; 72: 3–12. 25173806 10.1016/j.nbd.2014.08.025 PMC4253862

[49] Fortea J Pegueroles J Alcolea D , et al. APOE4 Homozygosity represents a distinct genetic form of Alzheimer’s disease. Nat Med 2024; 30: 1284–1291. 38710950 10.1038/s41591-024-02931-w PMC13310155

[50] Mahley RW Rall SC . Apolipoprotein E: far more than a lipid transport protein. Annu Rev Genomics Hum Genet 2000; 1: 507–537. 11701639 10.1146/annurev.genom.1.1.507

[51] Belloy ME Napolioni V Greicius MD . A quarter century of APOE and Alzheimer’s disease: progress to date and the path forward. Neuron 2019; 101: 820–838. 30844401 10.1016/j.neuron.2019.01.056 PMC6407643

[52] Corder EH Saunders AM Strittmatter WJ , et al. Gene dose of apolipoprotein E type 4 allele and the risk of Alzheimer’s disease in late onset families. Science 1993; 261: 921–923. 8346443 10.1126/science.8346443

[53] Yamazaki Y Zhao N Caulfield TR , et al. Apolipoprotein E and Alzheimer disease: pathobiology and targeting strategies. Nat Rev Neurol 2019; 15: 501–518. 31367008 10.1038/s41582-019-0228-7 PMC7055192

[54] Mendoza-Oliva A Zepeda A Arias C . The complex actions of statins in brain and their relevance for Alzheimer’s disease treatment: an analytical review. Curr Alzheimer Res 2014; 11: 967–985. 25274112

[55] Gentreau M Rukh G Miguet M , et al. The effects of statins on cognitive performance are mediated by low-density lipoprotein, C-reactive protein, and blood glucose concentrations. J Gerontol A Biol Sci Med Sci 2023; 78: 1964–1972. 37431946 10.1093/gerona/glad163 PMC10613010

[56] Jamshidnejad-Tosaramandani T Kashanian S Al-Sabri MH , et al. Statins and cognition: modifying factors and possible underlying mechanisms. Front Aging Neurosci 2022; 14: 975531. 10.3389/fnagi.2022.968039 PMC9421063 36046494

[57] Evans BA Evans JE Baker SP , et al. Long-term statin therapy and CSF cholesterol levels: implications for Alzheimer’s disease. Dement Geriatr Cogn Disord 2009; 27: 519–524. 19478483 10.1159/000221835

[58] Mach F Baigent C Catapano AL , et al. 2019 ESC/EAS guidelines for the management of dyslipidaemias: lipid modification to reduce cardiovascular risk. Atherosclerosis 2019; 290: 140–205. 31591002 10.1016/j.atherosclerosis.2019.08.014

[59] Leya M Stone NJ . Statin prescribing in the elderly: special considerations. Curr Atheroscler Rep 2017; 19: 47. 29019063 10.1007/s11883-017-0683-9

[60] Arnett DK Blumenthal RS Albert MA , et al. 2019 ACC/AHA guideline on the primary prevention of cardiovascular disease: executive summary. J Am Coll Cardiol 2019; 74: 1376–1414. 30894319 10.1016/j.jacc.2019.03.009 PMC8344373

[61] Grundy SM Stone NJ Bailey AL , et al. 2018 AHA/ACC/AACVPR/AAPA/ABC/ACPM/ADA/AGS/APhA/ASPC/NLA/PCNA guideline on the management of blood cholesterol: a report of the American College of Cardiology/American Heart Association task force on clinical practice guidelines. J Am Coll Cardiol 2019; 73: e285–e350. 10.1016/j.jacc.2018.11.003 30423393

[62] Carrero JJ Elinder CG . The Stockholm CREAtinine measurements (SCREAM) project: fostering improvements in chronic kidney disease care. J Intern Med 2022; 291: 254–268. 35028991 10.1111/joim.13418

[63] Runesson B Gasparini A Qureshi AR , et al. The Stockholm CREAtinine measurements (SCREAM) project: protocol overview and regional representativeness. Clin Kidney J 2016; 9: 119–127. 26798472 10.1093/ckj/sfv117 PMC4720196

[64] Religa D Fereshtehnejad SM Cermakova P , et al. Svedem, the Swedish dementia registry – a tool for improving the quality of diagnostics, treatment and care of dementia patients in clinical practice. PLoS One 2015; 10: e0116538. 10.1371/journal.pone.0116538 PMC4335024 25695768

[65] Wettermark B Hammar N Fored CM , et al. The new Swedish prescribed drug register – opportunities for pharmacoepidemiological research and experience from the first six months. Pharmacoepidemiol Drug Saf 2007; 16: 726–735. 16897791 10.1002/pds.1294

[66] Catapano AL Graham I De Backer G , et al. 2016 ESC/EAS guidelines for the management of dyslipidaemias. Eur Heart J 2016; 37: 2999–3058. 27567407 10.1093/eurheartj/ehw272

[67] Reiner Ž Catapano AL De Backer G , et al. ESC/EAS guidelines for the management of dyslipidaemias: the task force for the management of dyslipidaemias of the European Society of Cardiology (ESC) and the European atherosclerosis society (EAS). Eur Heart J 2011; 32: 1769–1818. 21712404 10.1093/eurheartj/ehr158

[68] Ludvigsson JF Svedberg P Olén O , et al. The longitudinal integrated database for health insurance and labour market studies (LISA) and its use in medical research. Eur J Epidemiol 2019; 34: 423–437. 30929112 10.1007/s10654-019-00511-8 PMC6451717

[69] Ludvigsson JF Andersson E Ekbom A , et al. External review and validation of the Swedish national inpatient register. BMC Public Health 2011; 11: 450. 21658213 10.1186/1471-2458-11-450 PMC3142234

[70] National Institute for Health and Care Excellence (NICE) . Cardiovascular disease: risk assessment and reduction, including lipid modification. NICE guideline NG238. London: NICE, 2023, Available at: https://www.nice.org.uk/guidance/ng238 (accessed 14 December 2023). 32200592

[71] Sundvall H Fastbom J Wallerstedt SM , et al. Use of statins in the elderly according to age and indication – a cross-sectional population-based register study. Eur J Clin Pharmacol 2019; 75: 959–967. 30826850 10.1007/s00228-019-02645-w

[72] Don-Doncow N Vanherle L Matthes F , et al. Simvastatin therapy attenuates memory deficits that associate with brain monocyte infiltration in chronic hypercholesterolemia. NPJ Aging Mech Dis 2021; 7: 20. 34349106 10.1038/s41514-021-00071-w PMC8338939

[73] Palmisano BT Zhu L Eckel RH , et al. Sex differences in lipid and lipoprotein metabolism. Mol Metab 2018; 15: 45–55. 29858147 10.1016/j.molmet.2018.05.008 PMC6066747

[74] Mombelli G Bosisio R Calabresi L , et al. Gender-related lipid and/or lipoprotein responses to statins in subjects in primary and secondary prevention. J Clin Lipidol 2015; 9: 226–233. 25911079 10.1016/j.jacl.2014.12.003

[75] Cangemi R Romiti GF Campolongo G , et al. Gender-related differences in treatment and response to statins in primary and secondary cardiovascular prevention: the never-ending debate. Pharmacol Res 2017; 117: 148–155. 28012963 10.1016/j.phrs.2016.12.027

[76] Stamerra CA Di Giosia P Ferri C , et al. Statin therapy and sex hormones. Eur J Pharmacol 2020; 890: 173618. 10.1016/j.ejphar.2020.173745 33227286

[77] Resnick SM Matsumoto AM Stephens-Shields AJ , et al. Testosterone treatment and cognitive function in older men with low testosterone and age-associated memory impairment. JAMA 2017; 317: 717–727. 28241356 10.1001/jama.2016.21044 PMC5433758

[78] Janowsky JS . The role of androgens in cognition and brain aging in men. Neuroscience 2006; 138: 1015–1020. 16310318 10.1016/j.neuroscience.2005.09.007

[79] Dobs AS Miller S Neri G , et al. Effects of simvastatin and pravastatin on gonadal function in male hypercholesterolemic patients. Metabolism 2000; 49: 115–121. 10647074 10.1016/s0026-0495(00)90938-7

[80] Dobs AS Schrott H Davidson MH , et al. Effects of high-dose simvastatin on adrenal and gonadal steroidogenesis in men with hypercholesterolemia. Metabolism 2000; 49: 1234–1238. 11016911 10.1053/meta.2000.7716a

[81] De Keyser CE De Lima FV De Jong FH , et al. Use of statins is associated with lower serum total and non–sex hormone-binding globulin–bound testosterone levels in male participants of the Rotterdam study. Eur J Endocrinol 2015; 173: 155–165. 26034077 10.1530/EJE-14-1061

[82] Mielke MM Zandi PP Sjögren M , et al. High total cholesterol levels in late life associated with a reduced risk of dementia. Neurology 2005; 64: 1689–1695. 15911792 10.1212/01.WNL.0000161870.78572.A5

[83] Benito-León J Vega-Quiroga S Villarejo-Galende A , et al. Hypercholesterolemia in elders is associated with slower cognitive decline: a prospective, population-based study (NEDICES). J Neurol Sci 2015; 350: 69–74. 25703278 10.1016/j.jns.2015.02.016

[84] Reitz C Tang MX Luchsinger J , et al. Relation of plasma lipids to Alzheimer disease and vascular dementia. Arch Neurol 2004; 61: 705–714. 15148148 10.1001/archneur.61.5.705 PMC2696387

[85] Wendell CR Zonderman AB Katzel LI , et al. Nonlinear associations between plasma cholesterol levels and neuropsychological function. Neuropsychology 2016; 30: 980–987. 27280580 10.1037/neu0000298 PMC5088056

[86] Williams DM Finan C Schmidt AF , et al. Lipid lowering and Alzheimer disease risk: a Mendelian randomization study. Ann Neurol 2020; 87: 30–39. 31714636 10.1002/ana.25642 PMC6944510

[87] de Oliveira FF Chen ES Smith MC , et al. Selected LDLR and APOE polymorphisms affect cognitive and functional response to lipophilic statins in Alzheimer’s disease. J Mol Neurosci 2020; 70: 1574–1588. 32474901 10.1007/s12031-020-01588-7

[88] de Oliveira FF Bertolucci PHF Chen ES , et al. Pharmacogenetic analyses of therapeutic effects of lipophilic statins on cognitive and functional changes in Alzheimer’s disease. J Alzheimers Dis 2022; 87: 359–372. 35311709 10.3233/JAD-215735

[89] de Oliveira FF Chen ES Smith MC , et al. Longitudinal lipid profile variations and clinical change in Alzheimer’s disease dementia. Neurosci Lett 2017; 646: 36–42. 28274859 10.1016/j.neulet.2017.03.003

