# Supplementary material for: Statins, cholesterol and cognition at the time of Alzheimer's disease diagnosis: A cross-sectional study from the Swedish registry for cognitive/dementia disorders
Source: J Alzheimers Dis Rep. 2025 Oct 3;9:25424823251385903. doi: 10.1177/25424823251385903 (PMC12495212; doi:10.1177/25424823251385903)
Supplement: sj-docx-1-alr-10.1177_25424823251385903 - Supplemental material for Statins, cholesterol and cognition at the time of Alzheimer's disease diagnosis: A cross-sectional study from the Swedish registry for cognitive/dementia disorders [file sj-docx-1-alr-10.1177_25424823251385903.docx]

**Supplemental Material**

**Statins, cholesterol and cognition at the time of Alzheimer's disease diagnosis: A cross-sectional study from the Swedish Registry for Cognitive/Dementia Disorders**

**Supplemental Table 1.** ATC codes for medications within six months before dementia diagnosis

| **Medications** | **ATC code** |
| --- | --- |
| RAAS | C09 |
| β-blocking agents | C07 |
| Calcium channel blockers | C08 |
| Diuretics | C03 |
| Nonsteroidal anti-inflammatory drugs | M01A |
| Peripheral vasodilators | C04 |
| Insulin | A10A |
| Other antidiabetics | A10B |
| Anticoagulants | B01AA, B01AB, B01AF, B01AE07 |
| Antiplatelets | B01AC |
| Antihypertensives | C03, C07-C09 |
| Cardiac drugs | C01 |
| Vasoprotective drugs | C05 |
| Vitamin D | A11CC |
| Antipsychotics | N05A |
| Anxiolytics | N05B |
| Hypnotics | N05C |
| Antidepressants | N06A |
| Statin | C10 |
| Simvastatin | C10AA01, C10BA02, C10BX01, C10BX04 |
| Atorvastatin | C10AA05, C10BA05, C10BX03, C10BX06, C10BX08, C10BX11, C10BX12, C10BX15 |

ATC: Anatomical Therapeutic Chemical; RAAS: Agents acting on the renin-angiotensin system.

**Supplemental Table 2.** ICD-10 codes for comorbidities within three years before dementia diagnosis

| **Comorbidities** | **ICD-10 code** |
| --- | --- |
| Hyperlipidemia | E780-E786 |
| Hypertension | I10-15 |
| Diabetes mellitus | E10-14 |
| Myocardial infarction | I21, I22, I252 |
| Congestive heart failure | I099, I110, I130, I132, I255, I420, I425-429, I43, I50, P290 |
| Type 1 diabetes mellitus | E10 |
| Type 2 diabetes mellitus | E11 |
| Chronic respiratory disease | J40-47, J60-67, J684, J701, J703 |
| Chronic kidney disease | E11.7, E12.2-E12.5, E12.7, E13.2–E13.5, E13.7, E14.2–E14.5, E14.7, N032-037, N052-057, N18, N19, N250, Z49, Z49.0, Z49.1, Z49.2, Z49.01, Z49.02, Z49.3, Z49.31, Z49.32, Z99.2, I77.0, Z94.0 |
| Cancer | C00-C97, D00-D49 |
| Atrial Fibrillation | I48 |
| Stroke | H341, I60, I61, I63, I64, I69 |
| Liver disease | K70-K77 |
| Ischemic heart disease | I20, I21, I22, I23, I24, I25 |
| Angina pectoris | I20 |
| Alcohol-related diseases | E244, F10, G312, G621, G721, I426, K292, K70, K860, O354, P043, Q860, T51, Y90, Y91, Z502, Z714 |
| Depression | F32, F33, F34.1, F34.8, F34.9, F38, F39 |
| Fracture | S12, S22, S32, S42, S52, S62, S72, S82, S92, M90 |

ICD-10: International Statistical Classification of Diseases and Related Health Problems, tenth revision.

**Supplemental Table 3.** ICD-10 codes for dementia diagnoses

| **Dementia** | **ICD-10 code** |
| --- | --- |
| Alzheimer's disease | F001, G301 |
| Mixed dementia | F002, G308 |
| Vascular dementia | F01 |
| Lewy Body dementia and Parkinson's disease with dementia | G318, F023 |
| Frontotemporal dementia | F020, G310 |
| Unspecified dementia | F03, G319 |
| Other dementia | F021, F022, F024, F028, F051, G311, G312 |

**Supplemental Table 4.** The association of baseline characteristics with use of statins

| **Variables** | **Simvastatin or atorvastatin (n=1028)** | |  |
| --- | --- | --- | --- |
|  | **OR (95 % CI)** | ***p*** | |
| Age at dementia diagnosis, y | 0.98 (0.97-0.99) | <0.001 | |
| Sex |  |  | |
| Male | REF |  | |
| Female | 0.89 (0.74-1.07) | 0.203 | |
| Educational level |  |  | |
| Completed compulsory education | REF |  | |
| Upper secondary | 0.74 (0.40-1.36) | 0.329 | |
| College/university | 1.25 (0.98-1.58) | 0.067 | |
| Missing | 1.17 (0.95-1.44) | 0.143 | |
| Coresident status |  |  | |
| Cohabiting | REF |  | |
| Living alone | 0.65 (0.34-1.21) | 0.174 | |
| Missing | 0.76 (0.63-0.92) | 0.005 | |
| Comorbidities |  |  | |
| Diabetes mellitus | 2.25 (1.79-2.82) | <0.001 | |
| Atrial fibrillation | 1.00 (0.76-1.31) | 0.986 | |
| Congestive heart failure | 0.64 (0.44-0.93) | 0.019 | |
| Stroke | 1.75 (1.25-2.45) | 0.001 | |
| Ischemic heart disease | 2.48 (1.83-3.35) | <0.001 | |
| Chronic kidney disease | 0.91 (0.74-1.13) | 0.389 | |
| Medication use |  |  | |
| Diuretics | 1.14 (0.92-1.40) | 0.231 | |
| Peripheral vasodilators | 2.08 (1.36-3.18) | 0.001 | |
| Vasoprotective drugs | 1.13 (0.80-1.58) | 0.495 | |
| β-blocking agents | 2.23 (1.84-2.71) | <0.001 | |
| Calcium channel blockers | 1.25 (1.02-1.53) | 0.031 | |
| RAAS | 2.30 (1.92-2.75) | <0.001 | |
| Antiplatelets | 2.74 (2.27-3.30) | <0.001 | |
| Hypnotics | 1.34 (1.08-1.68) | 0.009 | |
| Antipsychotics | 0.53 (0.33-0.83) | 0.006 | |
| Antidepressants | 1.19 (0.97-1.47) | 0.098 | |

RAAS: Agents acting on the renin-angiotensin system

**Supplemental Table 5.** Distribution of defined daily doses (DDD) of simvastatin and atorvastatin

| DDD | Simvastatin (n=818) | Atorvastatin (n=210) |
| --- | --- | --- |
| 0~1 | 579 (70.8) | 84 (40.0) |
| 1~2 | 218 (26.6) | 76 (36.2) |
| 2~3 | 21 (2.6) | 50 (23.8) |

Cumulative doses dispensed during the six months before dementia diagnosis were expressed as number of defined daily doses (DDD) in each package or dispensation. The DDD for each medication is defined by the World Health Organization. When multiple dispensations were used, their DDD were added.
